# Risk-sensitive foraging and the evolution of cooperative breeding and reproductive skew

**DOI:** 10.1186/1472-6785-8-2

**Published:** 2008-03-18

**Authors:** Hans J Poethke, Jürgen Liebig

**Affiliations:** 1 University of Würzburg, Field Station Fabrikschleichach, Glashüttenstrasse 5, D-96181 Rauhenebrach, Germany; 2 Arizona State University, School of Life Sciences and Center for Social Dynamics and Complexity, Tempe, AZ 85287-4501, USA

## Abstract

**Background:**

Group formation and food sharing in animals may reduce variance in resource supply to breeding individuals. For some species it has thus been interpreted as a mechanism of risk avoidance. However, in many groups reproduction is extremely skewed. In such groups resources are not shared equally among the members and inter-individual variance in resource supply may be extreme. The potential consequences of this aspect of group living have not attained much attention in the context of risk sensitive foraging.

**Results:**

We develop a model of individually foraging animals that share resources for reproduction. The model allows analyzing how mean foraging success, inter-individual variance of foraging success, and the cost of reproduction and offspring raising influence the benefit of group formation and resource sharing. Our model shows that the effects are diametrically opposed in egalitarian groups versus groups with high reproductive skew. For individuals in egalitarian groups the relative benefit of group formation increases under conditions of increasing variance in foraging success and decreasing cost of reproduction. On the other hand individuals in groups with high skew will profit from group formation under conditions of decreasing variance in individual foraging success and increasing cost of reproduction.

**Conclusion:**

The model clearly demonstrates that reproductive skew qualitatively changes the influence of food sharing on the reproductive output of groups. It shows that the individual benefits of variance reduction in egalitarian groups and variance enhancement in groups with reproductive skew depend critically on ecological and life-history parameters. Our model of risk-sensitive foraging thus allows comparing animal societies as different as spiders and birds in a single framework.

## Background

The evolution of group formation and cooperative breeding in animals has attracted considerable attention and there is a huge amount of literature with different approaches towards this phenomenon (reviewed in e.g. [1,2]). As group-living severely influences many aspects in the life of an organism (for a summary see [3]), its understanding requires a multifaceted approach. One of the factors that strongly affects the cost and benefits of group living is food availability. Group living may strongly increase the foraging success of individuals [3], though increasing inter-individual competition for food may also decrease individual food availability [4]. Foraging success plays a key role for the evolution of reproductive strategies [5]. The success of breeders often largely depends on the resources acquired (e.g. [6-10]) and group living is often associated with environmental constraints on resource acquisition [11-13].

In many species, a certain amount of resources is needed to start reproduction. This amount defines a reproduction threshold (see e.g. [14]). After having passed this reproduction threshold, additional food is often needed to successfully raise the offspring to independence. However, reproductive success as well as survival is not only affected by mean food availability but also by its variation in space and time [5,15-18]. The influence of variance in resource acquisition on foraging decisions has been extensively discussed in the context of risk sensitive foraging [16,18]. Models of risk sensitive foraging have shown that the mean amount of food an individual expects to acquire will strongly influence whether individuals should avoid risk and choose a more constant food supply or whether they should be risk prone and choose risky conditions with a high variance in foraging success [18-20]. When individuals form groups and share their food they may buffer fluctuations of their individual foraging success. Group formation with food sharing may thus reduce the variance in individual food supply and may be a mechanism of risk avoidance [21]. Consequently, models of risk sensitive foraging have been successfully applied to the problem of group formation [22].

Models of risk sensitive foraging are mostly focused on foraging for survival (for a summary see [23]). In general they predict that individuals should be risk averse if their mean foraging success surpasses a critical threshold needed for survival. Thus group formation should be restricted to situations with high mean as well as high variance of individual foraging success. This prediction has been confirmed by the observation that spiders form groups whenever mean foraging success is high [22,24,25]. Other examples are winter flocks of birds which reduce mortality risk by food sharing [26] or birds which become more and more risk prone with increasing mean foraging success [14].

It has been pointed out that risk-sensitive foraging may be favoured, not only for improved survival, but also for improved reproduction. However, risk-sensitive foraging for survival may require other strategic decisions than risk-sensitive foraging for reproduction [23,27]. Predictions strongly depend on the shape of the fitness function that relates food intake to reproductive success. Variance in foraging success can be advantageous for accelerating fitness functions, since individuals can disproportionately capitalize on high foraging success.

In egalitarian societies individuals equally share their resources for successful offspring production [28,29]. But resources are not necessarily shared equally between the members of a group. Physiological differences between individuals may influence the distribution of food and may result in reproductive skew. Reproductive skew may occur in societies as diverse as ants, bees, and mole rats [30]. However, reproductive skew may not only be a byproduct of competitive hierarchies in groups but may also be a mechanism that strongly increases the expected reproductive success of individuals within cooperative breeding groups. Although reproduction thresholds and the problem of offspring provisioning apply to solitary individuals as well as to cooperative breeders, the latter may respond differently to potential shortcomings in food availability: While solitary individuals are only able to invest in offspring production if their individual foraging success surpasses the reproduction threshold, cooperative breeders may direct the surplus food not needed for their own survival to reproductively dominant individuals or the dominant's offspring. This will enable the dominant individuals to pass the reproduction threshold more easily and produce offspring successfully. In many cooperatively breeding species helpers do not breed but contribute to raising the offspring of a few individuals that monopolize reproduction. This pattern occurs in vertebrates [1,31-33] as well as in insects [34-36]. With increasing reproductive skew within groups, dominant individuals may reach the reproduction threshold and have their offspring successfully raised at a lower per-capita individual foraging success.

Models of risk sensitive foraging analyze the influence of mean food availability and its variation on the benefits of variance reduction (e.g. by grouping and food sharing). They show that this benefit strongly depends on the specific form of the fitness function that relates food availability to breeding success or survival. However, when applied to groups these models strongly rely on the assumption that food is shared equally between the members of a group. It has not been analyzed so far, whether the predictions of these models will also hold when the focus is on reproduction in groups with significant reproductive skew. In the following we show that reproductive skew will severely affect the benefits of group formation.

## Results

### The resource-pooling model

Our model considerations are based on a stochastic model of foraging and resource allocation. We assume that animals search individually for food. Food collection is a stochastic process and the foraging success of individuals during a potential reproductive period follows a random distribution. We want to stress that we only consider the variation in the total foraging success that is needed to pass the reproduction threshold and to successfully raise offspring to independence within a reproductive period. We do not consider the daily variations in foraging success, since the consequences of group formation will be different for variation between days versus variation between reproductive periods. As foraging success varies between individuals and between reproductive periods it may be described by a probability density *P*(*x*, *μ*, *σ*), where *Pdx *gives the probability to successfully forage for the total amount of *x *(food items or energy gain) during one reproductive period if mean foraging success per period is *μ *and variance is *σ*^*2*^. For the sake of simplicity we will assume in the following examples that individual foraging success is approximately normally distributed. Thus, the probability that the foraging success of an individual within one reproductive period sums exactly up to the amount (x) of food is

$$P(x,\mu ,\sigma )\cdot dx=\frac{1}{\sigma \sqrt{2\pi }}e^{-\frac{{\left(x-\mu \right)}^{2}}{2\sigma^{2}}}\cdot dx$$

We also tested our model with other distributions (e.g. log-normal and Poisson distribution) and found similar results for all uni-modal types of distributions.

### The fitness function

We assume that the number of offspring an individual may successfully produce will depend on the amount of food it can acquire for itself and its offspring during a breeding season. The final reproductive success can thus be defined by a fitness function that relates the mean number of offspring produced (*F(x)*) to the amount of resources (*x*) an individual has collected during a breeding season. The simplest form for this function would be a linear relationship (e.g. [23]). However, such a relationship has two severe shortcomings: i) as no organism can produce indefinitely many offspring there will always be an upper limit to reproduction and ii) as a minimum of resources has to be invested to successfully produce and raise one single offspring there is a lower limit of resources necessary to start reproduction. Thus, due to the restrictive nature of a linear relationship, we use a more flexible function that enables investigation of a wider spectrum of ecological situations [37].

**Figure 1:**
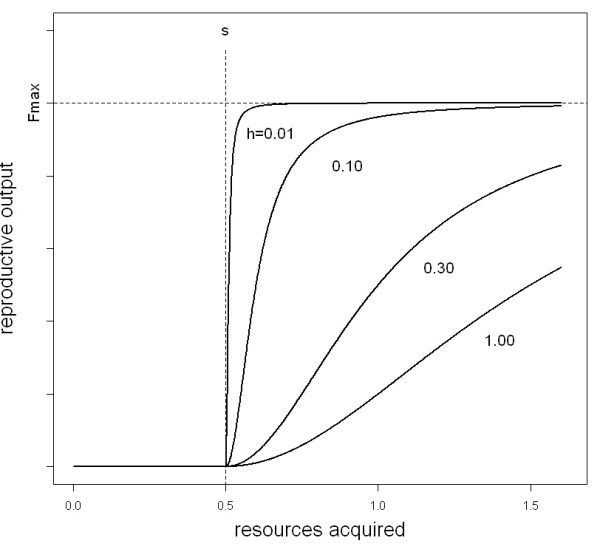
**The fitness function**. Relation between the amount of resources acquired and the expected reproductive success of individuals (equation 2) for a constant threshold value (*s *= 0.5) and four different values for the half-saturation constant (*h *= 0.01, 0.1, 0.3, 1.0).

Like Bednekoff [23] and Hurly [14] we assume that an individual may only reproduce if its foraging success is sufficient to provide the necessary resources to surpass a reproduction threshold (*s*). The threshold encompasses all cost incurred including those prior to reproduction of offspring, e.g. territory acquisition, nest building, mating costs, and it may consequently vary considerably between species. Once an individual has surpassed the reproduction threshold, the number of offspring it can raise should be dependent on the amount of resources it can further invest into the successful raising of offspring to adulthood. However, no matter how successful the individual will acquire resources, its physiology and the length of the season will set an upper limit to the number of offspring it can produce during one breeding season. All these conditions are easily met by a sigmoid fitness function (Fig. 1; see also [38] and [18]).

$$F(x)\{\begin{array}{ccc} 0 & if & x<s\\ F_{\max}\cdot \frac{{\left(x-s\right)}^{2}}{{\left(x-s\right)}^{2}+h^{2}} & if & x\geq s \end{array}$$

As can be seen from Fig. 1 this type of fitness function is able to cover a whole spectrum of different situations. For small values of the half-saturation constant (*h*) it will approach a step function (e.g. Fig. 1, *h *= 0.01). This describes a situation where the maximum reproductive output is easily reached once the reproduction threshold is surpassed. In this case batch size is almost independent of the amount of resources available. When the parameter (*h*) is relatively large compared to the range of plausible x-values (foraging success) the fitness function will increase almost linearly with the foraging success (*x*) after passing the reproduction threshold (e.g. Fig 1., *h *= 1.00). This would describe a situation when each additional offspring is relatively costly and individuals must invest substantial amounts of resources into raising additional offspring.

The expected number of offspring for a solitary individual (*E*_*solitary*_) may now be calculated as the weighted mean of its potential offspring numbers

$$E_{solitary}=\int_{0}^{\infty }P(x,\mu ,\sigma )\cdot F(F_{\max},h,s,x)\cdot dx$$

### Group formation

The amount of food acquired in groups with food pooling is simply the sum of individual food collection if there is no interaction between foraging individuals. We may thus simply multiply mean individual foraging success (*μ*) by group size (*n*) to get mean group income

*μ*_*group *_= *n*·*μ*

The same holds for the variance of group income

$$\sigma_{group}^{2}=n\cdot \sigma^{2}$$

If food is shared by all members of the group every single individual will get only a fraction (1n) of the total resources collected and consequently a group must collect (*n*·*s*) to start reproduction. The per capita reproductive output of a group is calculated as

Egroup=∫0∞P(x,n⋅μ,n⋅σ)⋅F(Fmax⁡,h,s,xn)⋅dx

However, when food is pooled in a group, it will not necessarily be evenly redistributed for reproduction or survival of group members. Groups may establish a reproductive hierarchy with some individuals reproducing more than others. The prime incentive for reproductive skew may be that sometimes there is not enough for all members to reproduce. If there is one group member that gets more than its proportional share of the group resources, this would allow it to reproduce, anyhow. For simplicity we may assume that there is only one reproductive individual in the group and we have complete skew i.e. the dominant breeder will get all resources that were collected for reproduction. Then this individual may successfully reproduce as soon as the group foraging success surpasses the threshold (*s*) and the expected per capita reproductive output of groups is

Eskew=1n⋅∫0∞P(x,n⋅μ,n⋅σ)⋅F(Fmax⁡,h,s,x)⋅dx

**Figure 2:**
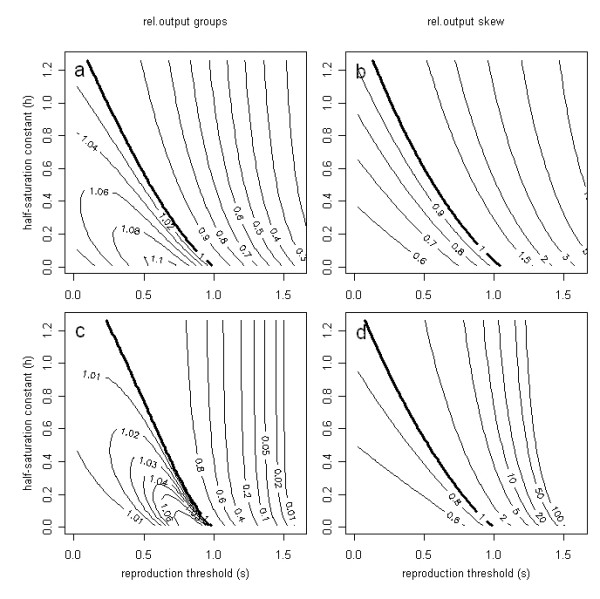
**Reproductive output of groups with and without skew**. Influence of the cost of reproduction (reproduction threshold, *s*), the reproductive potential of individuals (half saturation constant, *h*), and the standard deviation of individual foraging success (*σ*) on the relative reproductive success of groups (*N *= 2) without reproductive skew (a, *σ *= 0.5; c, *σ *= 0.2) and with reproductive skew (b, *σ *= 0.5; d, *σ *= 0.2). Integration of equations 3, 6 and 7 for mean foraging success *μ *= 1.

In the following we will restrict our model to a dyad (*n = 2*). As expected, the relative output of groups varies with the variance in the amount of resources individuals acquire during a season, with the reproduction threshold and with the shape of the fitness function (Fig. 2). However, most importantly all three factors influence egalitarian and reproductively skewed groups in a qualitatively different way.

### Egalitarian groups

Group formation with food pooling reduces the variance of foraging success for group members. In egalitarian groups, the beneficial effect of grouping on the reproductive success increases with increasing variance of the individual foraging success as illustrated by the comparison of Figs. 2a and 2c. However, this effect depends on the form of the fitness function which is determined by the half-saturation constant (*h*). Grouping is beneficial at low values of the half-saturation constant (*h*) and a low reproductive threshold (*s*). Species that normally pass the reproduction threshold and do not further invest in offspring rearing benefit most from forming egalitarian groups in our scenario. This is due to the fact that the fitness function approaches a step function for very small values of the half-saturation constant (*h*) (Fig. 1). In the area of foraging success (*μ*) above the reproductive threshold (*s*), the fitness function is thus decelerating in the major range of the foraging success. According to Jensen's inequality [39]), variance reduction with these conditions results in increased reproductive success for egalitarian groups (Figs. 2a and 2c).

With increasing half-saturation constant (*h*), the fitness function becomes more and more accelerating within the major range of the foraging success. According to Jensen's inequality [39], this will make variance reduction a bad strategy. Consequently, the relative reproductive success of individuals in egalitarian groups (compared to that of solitary individuals) decreases with increasing reproduction threshold (*s*) and with increasing half-saturation constant (*h*). On the other hand it will increase with increasing variance of total foraging success in the reproductive season.

### Groups with high reproductive skew

This pattern changes completely in groups with high reproductive skew, where all the resources for reproduction are invested into the dominant individual and its offspring (Figs. 2b and 2d). Reproduction will now be possible as soon as the group's combined foraging success provides enough resources to surpass the reproduction threshold of at least one individual.

The relative benefit of forming a group with high reproductive skew again depends on the amount of variance but with an opposite effect to egalitarian societies. Resource pooling in a group with skew is beneficial, whenever mean foraging success is below the reproduction threshold (*s*). With sufficient variance in foraging success some solitary individuals will be able to reproduce even if the mean foraging success is below the reproduction threshold.

However, the lower the variance in mean foraging success (*σ*) is, the smaller is the percentage of solitary individuals that would be above the reproduction threshold. The relative benefit of groups with food sharing and reproductive skew will, therefore, increase with decreasing variance (comparison of Figs. 2b and 2d).

The relative benefit of groups with reproductive skew also increases with increasing half-saturation constant (*h*), because this indicates that more resources are needed to successfully raise offspring after passing the reproduction threshold (*s*). With an increasing need of additional food along an increasing (*h*), the number of solitary individuals that are able to provide these resources decreases.

### Joining conditions

Evidently it may pay to join a group when per capita reproductive output in groups is larger than that of solitary individuals. But this will only hold for all group members if the reproductive output is equally shared between them or if relatedness between group members is high. For dyads with reproductive skew the direct and indirect fitness benefits of individuals strongly depend on their role and relatedness in the group (see also [40]). Both group members may have a chance to become reproductively dominant. This chance may vary between individuals and we denote the probability to become the reproductively dominant individual as (*d*). The expected gain in direct fitness for reproductively dominant individuals in a dyad (*n *= 2) is: 2·*E*_*skew*_. However, if the individual does not succeed in getting the reproductive role and stays as helper it can nevertheless receive indirect fitness benefits via its relatedness to the reproducing individual. This expected gain in indirect fitness equals: *r*·2·*E*_*skew*_. Thus in groups with absolute skew these fitness components sum up to the expected fitness gain of an individual: [*d *+ (1 - *d*)·*r*]·2·*E*_*skew*_. If an individual remains solitary the equivalent benefit would be (1 + *r*)·*E*_*solitary*_. Thus an individual should only join a group with skew if

EskewEsolitary>1+r2⋅[r⋅(1−d)+d]

If both group members have the same chance to become the reproductive individual (*d *= 0.5) there is no difference in the expected fitness for both individuals and from equation 8 follows that joining is independent of relatedness and group formation pays whenever *E*_*skew *_> *E*_*solitary*_. The same joining condition results, if both individuals are clones (*r *= 1). In this case the joining condition becomes independent of *d*. For *d *= 0.5 or *r *= 1 the advantage of joining a group thus only depends on the parameters determining the effect of resource pooling (Figs. 2b and 2d).

If group members are completely unrelated (*r = 0*) there must be a chance to become the reproductively dominant group member to make group formation attractive. Both group members should have a chance to receive the benefit of the accumulated resources. This is possible when food is shared indirectly by investing into a resource, such as a nest structure, that both group members can win. Following equation 8, forming a group with a completely unrelated partner will pay whenever 2*d*·*E*_*skew *_> *E*_*solitary*_. If an individual has a 10% chance only (*d *= 0.1) of becoming the reproductive, the per capita success of groups must be five times that of solitary individuals (*E*_*skew *_> 5·*E*_*solitary*_) to make group formation profitable. It can be seen from Figs. 2b and 2d that groups can fulfil this condition when the reproduction threshold is much higher than the mean individual foraging success, when variance in foraging success is small, or when the half saturation constant (*h*) of the fitness function is large.

In groups with skew it is obviously always better to become reproductive than to become a helper. Consequently group formation will depend on the motivation of the helping individual whenever the chances to become reproductively dominant differ between individuals. In the most extreme case the roles are fixed before the group is formed. Now the joining condition for the obligate helper (*d *= 0) strongly depends on its relatedness (*r*) to the dominant breeder. If helpers are siblings of the dominant (*r *= 0.5) joining will be profitable whenever *E*_*skew *_> 1.5·*E*_*solitary*_. This condition is fulfilled for a broad range of model parameters and even for first cousins (*r *= 0.25) we get a joining condition (*E*_*skew *_> 2.5·*E*_*solitary*_) that may make joining a group attractive for helpers even if the reproduction threshold is below the mean amount of resources an individual can acquire (Fig. 2d).

## Discussion

The results of our model analysis clearly demonstrate that the benefit of group formation and sharing resources for reproduction will depend critically on the amount of reproductive skew in a group. It is interesting to note that the introduction of skew in a group of communal breeders will qualitatively change the influence of the cost of reproduction (*s*), the half saturation constant (*h*) of the fitness function, and the variance (*σ*) of the foraging success of individual animals on the relative reproductive success of individuals in groups. This effect is readily explained by opposing influences of food pooling and reproductive skew on the variance in individual food availability.

Group formation and pooling of resources may be seen as a very general mechanism of variance reduction. When individuals pool the resources they have collected individually and evenly redistribute pooled resources between group members they can severely reduce the variance in the amount of food available to group members. This effect increases with increasing group size and the larger the group the smaller the variance in individual food availability will be.

However, while the temporal variability in the per capita amount of resources available in a group will usually decline with increasing group size [21], inter-individual variance in the amount of resources consumed by group members will not necessarily do so. In groups with reproductive skew resources are not shared equally between group members and inter-individual variance in food consumption may be strongly increased by skewed use of resources.

Following Jensen's inequality [18,39], the reduction of variance in disposable resources makes the formation of egalitarian groups profitable whenever the fitness function (Fig. 1) is upward convex. For our model this will be the case when the cost of reproduction (*s*) is small (relative to the expected amount of resources acquired, *s < m*) and when the maximum reproductive output of individuals is easily reached (small half saturation constant *h*). This prediction is corroborated by observations on colonial spiders [25], which form colonies whenever prey capture exceeds a threshold level. Thus, the decrease of per capita reproductive output in groups without skew with increasing cost of reproduction and increasing half saturation constant (Fig. 2a and 2c) is readily explained by Jensen's inequality.

On the other hand, group formation with reproductive skew is beneficial whenever reproduction is costly (*s > m*) and maximum reproduction is not easily reached (*h *sufficiently high). In groups with reproductive skew resources are combined to allow at least one individual to successfully raise its offspring while the other individuals do not reproduce directly but become helpers. Such helping behaviour is common among vertebrates [31,41,42] and ubiquitous in social insects [34,35]. Across carnivores, cooperative breeding in high skew societies is associated with high reproductive costs [43]. An increase of helping behaviour with increasing cost of reproduction has also been observed in Pied Kingfishers [44]). In this species pairs generally breed successfully at Lake Naivaha but are dependent on helpers at Lake Victoria, where prey is smaller, hunting success is lower and distance to prey is larger [44]. making provisioning of young much more expensive in the Lake Victoria colony [45]. While helping behaviour is a facultative strategy for Pied Kingfishers it may become fixed for species like White-Winged Choughs and the apostlebird where the reproduction threshold is so high, that helpers finally become essential for breeding [46,47]. All these examples highlight the importance of reproductive cost for the evolution of reproductive skew and totally comply with our model predictions.

Reproductive skew models explain how the assumed benefits of group formation are shared among dominant and subordinate individuals of a group [40,48-51]. Based on group benefits, relatedness and the potential cost of conflicts they explain the evolution of egalitarian as well as high skew societies. However, reproductive skew models do not intend to explain the generation of group benefits and their relation to ecological conditions. This is the primary goal of our model. We suggest that the transition from communal breeding to high skew groups occurs along a gradient of resource limitation and resource predictability. Under poor environmental conditions when the mean amount of resources an individual can invest into reproduction lays below the reproduction threshold this threshold will hardly be reached by solitary individuals. In such situations solitary individuals may profit from high variance in resource availability. But conditions with low and rather constant supply of resources will support the evolution of high skew societies. Our model clearly demonstrates that it may be advantageous for the members of groups to focus resources on a single breeder. In fact this is the ancestral and most common form of social organization in most social insects (e.g. [34,52]). Egalitarian social insects on the other hand are comparatively rare (but see [53]).

According to our model, living in groups always yields a higher per capita reproductive output than solitary life. Whether egalitarian groups or groups with high reproductive skew are the better choice simply depends on reproductive cost and variance of individual foraging success. However, this does not necessarily mean that all species should live in groups under all conditions. On the first hand we have to keep in mind that evolution is driven by individual selection rather than group selection. We have shown that helpers in groups with high reproductive skew must either be related to the reproductively dominant individual or they must have a chance to become the reproductively dominant individual themselves. Dependent on relatedness (*r*) and the chance to become dominant (*d*) the range of possible parameter values that predict the formation of groups with skew may be much smaller than the range that shows increased group productivity (equ. 8).

Though the control of variance in resource supply may be a very general and important benefit for groups that share resources there are further benefits of grouping. Grouping may increase mean foraging success since groups may serve as information centres on the availability of attractive food patches [54] and group members may be more efficient in hunting prey [55,56]. Group living may increase the survival of group members as groups can be more vigilant [57], better defended [58], and the risk of being attacked may be reduced for single group members [59]. All of these mechanisms may increase the benefit of group formation and their relative importance will vary enormously between species. On the other hand there are a number of potential costs associated with the formation of groups (for a summary see [3]). Individuals living in groups may be more easily detected by predators [59]. Individuals in groups may exploit resources more readily resulting in lower per capita consumption [60,61]. The formation of a group may result in conflicts over the social status of group members or over the distribution of group resources [25,62]. Such conflicts may incur severe costs. Particularly when groups become large the amount of resources acquired by individual members may decrease with increasing group size, as resources in the vicinity of the nest become scarce and the distances to be covered by foraging individuals increase [63]. All these additional costs may reduce the benefit of group formation significantly and may make solitary life the better strategy.

In our model we considered two effects of group formation, only: the reduction in the variance of food available to group members and (in case of reproductive skew) the uneven distribution of food between individuals. We assume that pooling of resources influences reproduction only. But the amount of food acquired not only determines the production of offspring but also the survival of an individual [64]. Thus, two thresholds determine the success of animals: the amount of food needed to survive and that needed to successfully produce offspring [14]. Both thresholds should have a major impact on foraging decisions. Arguments concerning the group advantages for survival would follow the same line as those concerning the group advantages for reproduction. When the amount of resources needed for survival is relatively small (compared to mean resource availability) most individuals will survive. However, for high variance of resource availability not all individuals will succeed to acquire sufficient resources for survival. In this case variance reduction by group formation and pooling of resources may substantially reduce mortality from starvation. This additional benefit may strongly increase the benefit of grouping shown in figs. 2a and 2c. However, we have to keep in mind that a thorough understanding of the joint influence of mortality and offspring production on the evolution of group formation and food sharing would require imbedding our model into a theoretical framework that allows taking care of the demographic ecological consequences of life history modifications [65]. Adaptive dynamics [66] may be an adequate theoretical concept to do this.

## Conclusion

Resource sharing in groups is a general mechanism of variance reduction while reproductive skew on the other hand allows increasing inter-individual variation in the amount of resources available for reproduction. Thus, in resource sharing groups the strength of reproductive skew allows controlling the variance in resource availability. This will allow social groups to behave either more risk averse or more risk prone dependent on the mean foraging success (*μ*), the reproductive threshold (*s*), and on the specific form of the fitness function (*h*). Consequently groups that can adjust the amount of reproductive skew will always be able to achieve higher per capita reproductive output than solitary individuals. According to our model environments with low individual foraging success and high reproduction cost will favour the evolution of reproductive skew, while large variance of foraging success, and relatively low reproduction cost will favour the formation of egalitarian groups. Although our model does not explain all forms of sociality and risk-sensitive foraging is only one factor among others, our model allows comparing a broad range of animal societies in a single framework. While classical reproductive skew models are focussed on the analysis of conflicts arising from uneven distribution of resources in groups we concentrate on mechanisms that generate group benefits for egalitarian or high-skew groups. Our approach relates group benefits to major ecological factors and life history components. We show that group benefits are strongly affected by variance in foraging success within a reproductive season, constraints on reproduction determined by the reproduction threshold, and investment in offspring production after passing this threshold.

## Methods

We derived an analytical model for the breeding success of solitary individuals, individuals that breed in groups and share food equally, and individuals that breed in groups with a pronounced bias in food distribution. Breeding success was modelled as a set of integral equations. The implementation was conducted as a numerical integration of these equations (eqns. 3, 6 and 7). Model equations were integrated using the programming language R version 2.3.1 [67].

## Authors' contributions

Both authors developed the basic idea of this paper and the structure of the model. HP developed the mathematical model, implemented it, carried out the numerical calculations and led the drafting of the manuscript. Both authors read and approved the final manuscript.
